# Toward actionable interventions in human aging (12th ARDD meeting, 2025)

**DOI:** 10.18632/aging.206368

**Published:** 2026-04-06

**Authors:** Aleksandr Dekan, Sierra Lore, Ye Eun Yoon, Aisyah Sjöholm, Alexander Tyshkovskiy, Alexey Terskikh, Ana Maria Cuervo, Anastasia Georgievskaya, Andrea Heinz, Andrei Seluanov, Andrew Adams, Andy P. Tsai, Anna Murray, Anne Brunet, Anne Marije van Harten, Avi Spier, Ben Shenhar, Bente Klarlund Pedersen, Berthil Clasen, Björn Schumacher, Brian K. Kennedy, Charlotte Suetta, Christiane Volbracht, Christoph Kuppe, Clive Cookson, Collin Y. Ewald, Cristiana Banila, Daisy Cai, Dario Riccardo Valenzano, Dudley W. Lamming, Edward Rudnic, Elias Bou Samra, Emma Teeling, Eric Verdin, Evandro Fei Fang, Fabrizio d’Adda di Fagagna, Fedor Galkin, Garri Zmudze, Heinrich Jasper, In Hwa Jang, Jing-Dong Jackie Han, Jamie Justice, Jan H. J. Hoeijmakers, Javier María Peralta Ramos, Jean-Marc Lemaitre, Jeroen Aerssens, Joe Betts-LaCroix, John C. Newman, Juan Carlos Izpisua Belmonte, Kazuto Kawamura, Kennedy Matsagas Schaal, Kitsu Egerton, Kotb Abdelmohsen, Kristen Fortney, Lars Hartenstein, Laura Niedernhofer, Lida Katsimpardi, Lisa Melton, Lotte Bjerre Knudsen, Luigi Ferrucci, Lykke Sylow, Marco Demaria, Marissa J. Schafer, Marta Guasch-Ferré, Martin Borch-Jensen, Maxim Kholin, Michael Antonov, Michael Levitt, Michela Deleidi, Miriam Merad, Morten Meldal, Mourad Topors, Murali Venkatesan, Nathan K. LeBrasseur, Niklas Anzinger, Peter Fedichev, Paul Kohlhaas, Peter J. Mullen, Priya Singhal, Rafael de Cabo, Riekelt H. Houtkooper, Rusty Montgomery, Ryan Smith, Sharon Rosenzweig-Lipson, Sergey Jakimov, Steve Horvath, Steven Austad, Thomas A. Rando, Todd White, Tom Zuber, Tomaz Rozmaric, Tony Wyss-Coray, Tuomas Tammela, Vadim N. Gladyshev, Vanessa V. C. Sinatti, Weilan Wang, William Harborne, Xiaodong Liu, Xing Li Wang, Yousin Suh, Yuta Lee, Zane Koch, Alex Zhavoronkov, Morten Scheibye-Knudsen, Daniela Bakula

**Affiliations:** 1 Department of Cellular and Molecular Medicine, University of Copenhagen, Copenhagen, Denmark; 2 Buck Institute for Research on Aging, Novato, CA 94945, USA; 3 Insilico Medicine AI, Abu Dhabi, United Arab Emirates; 4 Division of Genetics, Brigham and Women’s Hospital, Harvard Medical School, Boston, MA 02115, USA; 5 Harry Perkins Institute of Medical Research and University of Western Australia, Perth, WA, Australia; 6 Einstein Institute for Aging Research, Albert Einstein College of Medicine, New York, NY 10461, USA; 7 HautAI OU, Tallinn, Estonia; 8 Department of Pharmacy, LEO Foundation Center for Cutaneous Drug Delivery, University of Copenhagen, Copenhagen, Denmark; 9 Department of Biology and Medicine, University of Rochester, Rochester, NY 14609, USA; 10 Eli Lilly and Company, Indianapolis, IN 46225, USA; 11 Department of Neurology and Neurological Sciences, Stanford University, Stanford, CA 94305, USA; 12 Department of Clinical and Biomedical Science, Faculty of Health and Life Science, University of Exeter, Exeter, UK; 13 Department of Genetics, Stanford University, Stanford, CA 94305, USA; 14 Apollo Health Ventures, Luxembourg City, Luxembourg; 15 Novartis, Cambridge, MA 02139, USA; 16 Department of Molecular Cell Biology, Weizmann Institute of Science, Rehovot, Israel; 17 Centre for Physical Activity Research, Rigshospitalet, University of Copenhagen, Copenhagen, Denmark; 18 Institute for Genome Stability in Ageing and Disease, Cologne Cluster of Excellence on Aging and Aging-Associated Diseases (CECAD), University and University Hospital of Cologne, Cologne, Germany; 19 Centre for Healthy Longevity, National University Health System, Singapore, Singapore; 20 Healthy Longevity Translational Research Programme, Yong Loo Lin School of Medicine, National University of Singapore, Singapore, Singapore; 21 Department of Geriatric and Palliative Medicine, Copenhagen University Hospital, Bispebjerg and Frederiksberg, University of Copenhagen, Copenhagen, Denmark; 22 Neuroscience, H. Lundbeck A/S, Valby, Denmark; 23 Division of Nephrology and Clinical Immunology, RWTH Aachen University, Medical Faculty, Aachen, Germany; 24 Financial Times, London, UK; 25 Novartis Biomedical Research, Diseases of Aging and Regenerative Medicine, Basel, Switzerland; 26 Mitra Bio, Translation and Innovation Hub, London, UK; 27 B Capital, Manhattan Beach, CA 90266, USA; 28 Leibniz Institute on Aging, Fritz Lipmann Institute (FLI), Jena, Germany; 29 Department of Medicine, Division of Endocrinology, Diabetes, and Metabolism, University of Wisconsin-Madison, Madison, WI 53706, USA; 30 Maxwell Biosciences, Austin, TX 78738, USA; 31 L’Oréal Research and Innovation, Aulnay-sous-Bois, France; 32 School of Biology and Environmental Science, University College Dublin, Dublin, Ireland; 33 Department of Clinical Molecular Biology, University of Oslo, Oslo, Norway; 34 IFOM, Milan, Italy and IGM-CNR, Pavia, Italy; 35 LongeVC, Washington, DC 20036, USA; 36 Genentech Inc., South San Francisco, CA 94080, USA; 37 Masonic Institute on the Biology of Aging and Metabolism (MIBAM), University of Minnesota, Minneapolis, MN 55455, USA; 38 Center for Quantitative Biology, Peking University, Beijing, China; 39 XPRIZE Healthspan, Los Angeles, CA 90401, USA; 40 Department of Molecular Biology, Erasmus Medical Center, Rotterdam, and Princess Maxima Center for Pediatric Oncology, ONCODE Institute, Utrecht, The Netherlands; 41 Department of Brain Sciences, Weizmann Institute of Science, Rehovot, Israel; 42 Institute for Regenerative Medicine and Biotherapy (IRMB), University of Montpellier, INSERM, Montpellier, France; 43 Rejuvenate Biomed, Diepenbeek, Belgium; 44 Retro Biosciences, Redwood City, CA 94115, USA; 45 Division of Geriatrics, University of California San Francisco, San Francisco, CA 94118, USA; 46 Altos Labs, San Diego Institute of Science, San Diego, CA 92121, USA; 47 Max Planck Institute for Biology of Ageing, Cologne, Germany; 48 Rejuve Bio, Orange, CA 92867, USA; 49 Baillie Gifford and Co., Edinburgh, UK; 50 National Institute on Aging, National Institutes of Health (NIH), Baltimore, MD 21224, USA; 51 BioAge Labs, Richmond, CA 94804, USA; 52 McKinsey Health Institute, New York, NY 10007, USA; 53 Nature Biotechnology, New York, NY 10013, USA; 54 Novo Nordisk A/S, Måløv, Denmark; 55 Department of Biomedical Sciences, Faculty of Health and Medical Sciences, University of Copenhagen, Copenhagen, Denmark; 56 European Research Institute for the Biology of Ageing (ERIBA), University Medical Center Groningen (UMCG), University of Groningen (RUG), Groningen, The Netherlands; 57 Department of Physiology and Biomedical Engineering, Mayo Clinic, Rochester, MN 55905, USA; 58 Novo Nordisk Center for Basic Metabolic Research and Department of Public Health, University of Copenhagen, Copenhagen, Denmark; 59 Department of Public Health, University of Copenhagen, Copenhagen, Denmark; 60 Gordian Biotechnology, San Francisco, CA 94080, USA; 61 Gero, Singapore, Singapore; 62 Deep Origin, Palo Alto, CA 94080, USA; 63 Department of Structural Biology, Stanford University, Stanford, CA 94304, USA; 64 Institute Imagine, INSERM UMR1163, Paris, France; 65 Precision Immunology Institute, Icahn School of Medicine at Mount Sinai, New York, NY 10029, USA; 66 Department of Chemistry, University of Copenhagen, Copenhagen, Denmark; 67 Repair Biotechnologies, Syracuse, NY 13210, USA; 68 Danaher Ventures, Danaher Corporation, Washington, DC 20037, USA; 69 Robert and Arlene Kogod Center on Aging, Mayo Clinic, Rochester, MN 55905, USA; 70 Infinita City, Prospera, Roatán, Honduras; 71 Molecule AG, Basel, Switzerland; 72 Department of Immunology and Immune Therapeutics, Keck School of Medicine, Leonard Davis School of Gerontology, University of Southern California, Los Angeles, CA 90033, USA; 73 Biogen, Cambridge, MA 02142, USA; 74 Laboratory Genetic Metabolic Diseases, Amsterdam Gastroenterology, Endocrinology and Metabolism Institute, Amsterdam UMC, University of Amsterdam, Amsterdam, The Netherlands; 75 TruDiagnostic Inc., Lexington, KY 40503, USA; 76 Life Biosciences, Boston, MA 02116, USA; 77 Department of Biology, University of Alabama at Birmingham, Birmingham, AL 35294, USA; 78 UCLA Broad Stem Cell Research Center, Los Angeles, CA 90033, USA; 79 The Thalion Initiative, Boston, MA 02115, USA; 80 Zuber Lawler, Los Angeles, CA 90067, USA; 81 Ludwig Boltzmann Institute for Traumatology, Vienna, Austria; 82 Department of Neurology and Neurological Sciences, Stanford University, Stanford, CA 94304, USA; 83 Cancer Biology and Genetics Program, Sloan Kettering Institute, Memorial Sloan Kettering Cancer Center, New York, NY 10065, USA; 84 Aptah Bio Inc., San Carlos, CA 94070, USA; 85 LongGame Ventures, London, UK; 86 School of Life Sciences, Westlake University and Westlake Laboratory of Life Sciences and Biomedicine, Hangzhou, China; 87 Fosun Pharma, Shanghai, China; 88 Department of Obstetrics and Gynecology, Columbia University, New York, NY 10032, USA; 89 Department of Genetics and Development, Columbia University, New York, NY 10032, USA; 90 Accelerated Biosciences Corp., Philadelphia, PA 19104, USA; 91 Program in Bioinformatics and Systems Biology, University of California, San Diego, La Jolla, CA 92093, USA; 92 Insilico Medicine US, Cambridge, MA 02138, USA; 93 Insilico Medicine Hong Kong, Hong Kong SAR, China

**Keywords:** aging, longevity, geroscience, biomarkers, rejuvenation

## Abstract

The 12th Aging Research and Drug Discovery (ARDD) meeting convened at the University of Copenhagen, presenting a comprehensive overview of recent advancements in the biology of aging. A central theme across sessions was the field’s gradual shift from descriptive, correlational studies to mechanistic understandings enabling the engineering of personalized therapeutic interventions aimed at extending human healthspan. Key discussions highlighted the convergence of multiple disciplines. Presentations detailed how fundamental biological insights are being integrated with artificial intelligence and machine learning platforms for accelerated target identification and drug development. Furthermore, the development and application of novel preclinical research models were presented as critical for improving the translational pipeline to human clinical trials. Scientific discourse has advanced from cataloging the established hallmarks of aging to identifying and modulating the specific molecular mechanisms that regulate them. This focus is predicated on the hypothesis that aging is not solely a result of stochastic damage accumulation but may be a tractable, modifiable, and potentially reversible biological process amenable to intervention. This report summarizes the principal research directions and conceptual frameworks presented at the conference.

## Is biological age reversible?

**Figure 1:**
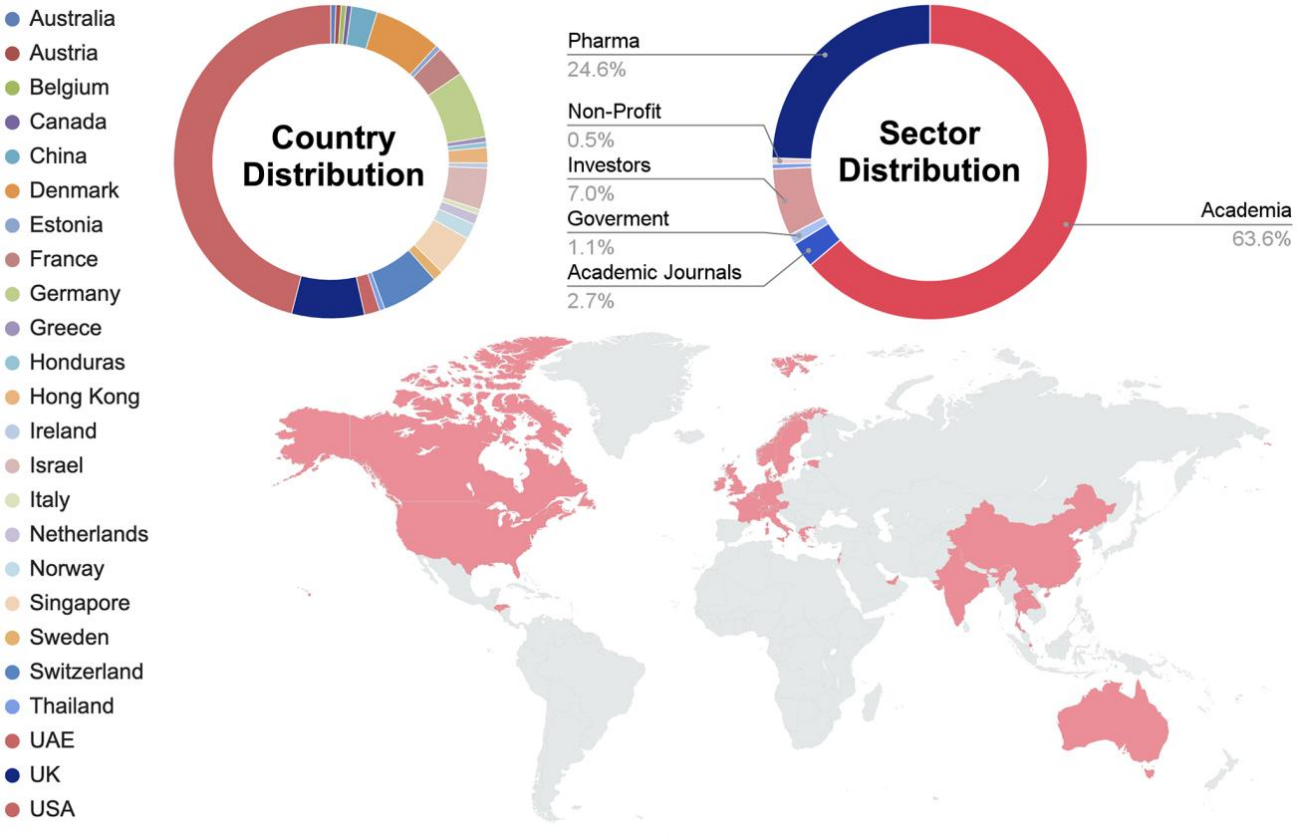
Statistics and Geography of ARDD 2025 speakers.

**Figure 2:**
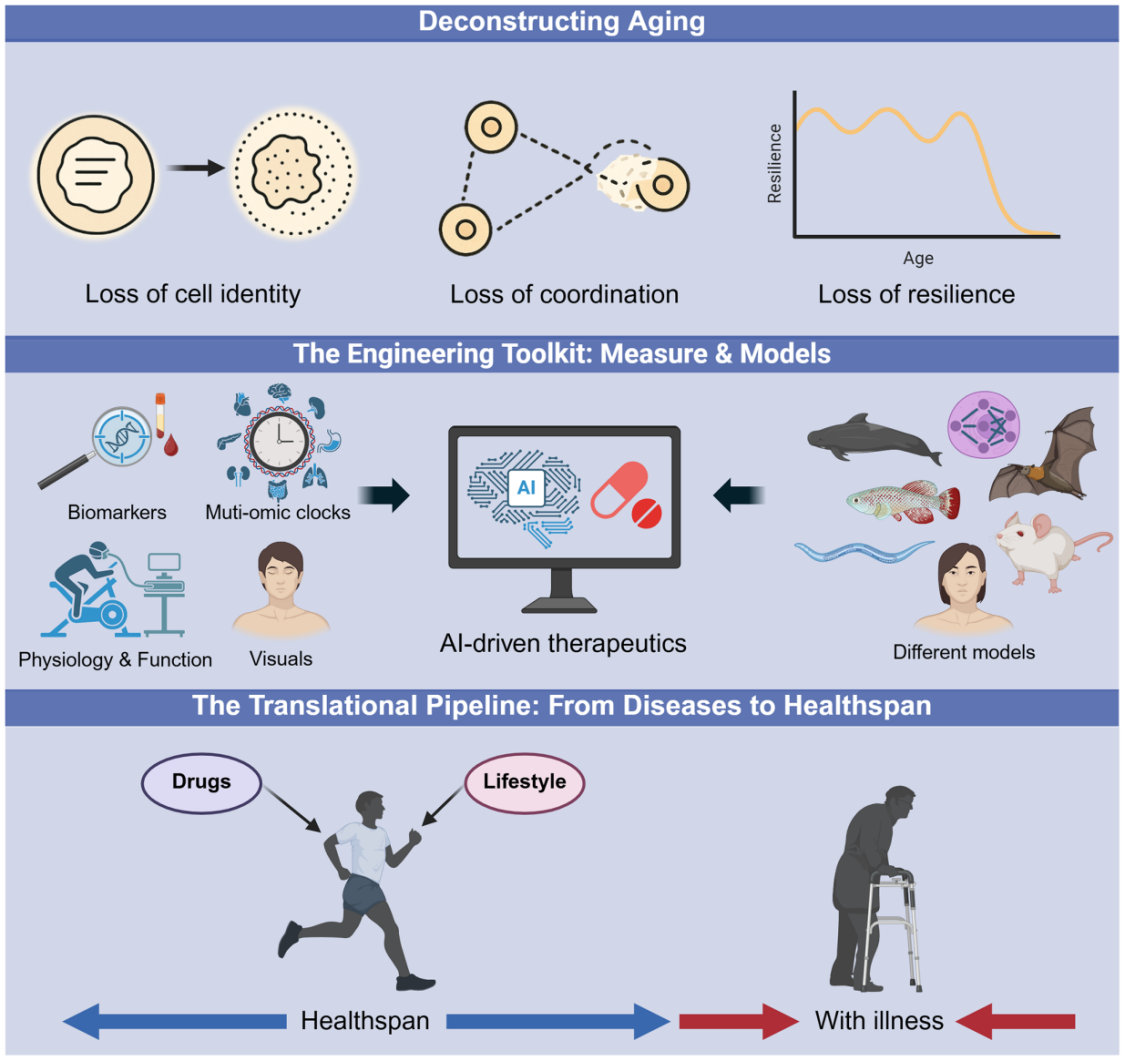
**The roadmap to actionable longevity interventions.** An overview of the conceptual frameworks presented at the 12th ARDD meeting. (Top) Deconstructing Aging: Moving beyond stochastic damage to identify modifiable drivers of aging, such as the loss of cellular identity, coordination, and systemic resilience. (Middle) The Engineering Toolkit: The integration of precise measurement tools, including organ-specific multi-omic clocks and biomarkers, with diverse preclinical models and AI platforms to accelerate therapeutic design. (Bottom) The Translational Pipeline: The strategic shift from reactive disease management to proactive healthspan extension, utilizing novel drugs and lifestyle interventions to maintain youthful function.

The 12th Aging Research and Drug Discovery (ARDD) meeting brought speakers from all over the world, representing the comprehensive spectrum of the aging field, including academic research, industrial development, investment capital, and media coverage (Figure 1). A synopsis of the meeting’s proceedings is presented in Figure 2.

Among the many topics covered, a central question dominated the mechanistic sessions of the meeting: can we reverse biological age? Several presentations provided a nuanced “yes,” pointing to the epigenome as a master controller of cellular identity and function.

### The reversible nature of epigenetic drift

The idea that aging is a reversible loss of youthful epigenetic information was brought into conversation. Jean-Marc Lemaitre (University of Montpellier, Montpellier, France) raised a key mechanistic question: is pluripotency necessary for rejuvenation? His work in a progeric mouse model demonstrated that a single, transient reprogramming event administered early in life extends healthy lifespan by 15%, preserving muscle integrity and preventing osteoarthritis, osteoporosis and lung fibrosis, suggesting the establishment of a persistent, youthful epigenetic state [1]. However, this intervention failed when applied late in life, suggesting that the system’s starting point is critical. Providing a unifying theory, Juan Carlos Izpisua Belmonte (Altos Labs, San Diego, CA, USA) framed aging as a progressive loss of cellular identity he termed “mesenchymal drift,” a process in which specialized cells shift toward more mesenchymal-like or hybrid states, driving tissue dysfunction and disease [2]. He argued that partial reprogramming may be particularly potent because it directly reverses this drift, a hypothesis that is increasingly well-supported. In a proof-of-concept study, his team rejuvenated aged kidneys ex vivo using reprogramming factors and successfully transplanted them into rats, bringing the vision of organ regeneration closer to clinical feasibility.

But does rejuvenation require treating every cell? In unpublished work, Thomas Rando (University of California Los Angeles, Los Angeles, CA, USA) revealed that local epigenetic reprogramming has profound systemic effects. By expressing Yamanaka factors only in the non-dividing muscle fibers of mice, his lab not only rejuvenated the muscle but also observed rejuvenation in distant organs like the spleen and kidney. This finding reframes muscle as an organ capable of communicating a pro-youthful state throughout the body, offering therapeutic potential where targeting one accessible tissue could yield systemic rejuvenation consistent with previous research [3].

Sharon Rosenzweig-Lipson (Life Biosciences, Boston, MA, USA) detailed their partial epigenetic reprogramming platform, using three Yamanaka factors (Oct4, Sox2, and KLF4, excluding c-Myc). She reported that recent Good Laboratory Practice (GLP) toxicology studies and results from a non-human primate nonarteritic anterior ischemic optic neuropathy (NAION) model showed that the therapy can reverse disease-associated DNA methylation patterns in retinal ganglion cells. Based on these findings, a first-in-human clinical trial for glaucoma and NAION is planned for the first quarter of 2026. Rosenzweig-Lipson also expanded the platform’s application by presenting new data from a metabolic dysfunction-associated steatohepatitis (MASH) mouse model, which demonstrated significant improvements in liver enzymes and steatosis without any corresponding body weight change, pointing to a direct, organ-specific effect.

Challenging conventional wisdom, Tuomas Tammela (Sloan Kettering Institute, New York, NY, USA) presented a surprising connection between aging and cancer. Contrary to expectations, his lab found that aged mice develop significantly fewer and smaller lung adenocarcinomas than their younger counterparts [4]. The protective mechanism was traced to an epigenetic program in aged alveolar stem cells, driven by DNA hypomethylation, which leads to high expression of the transcription factor NUPR1. This, in turn, creates a state of iron deficiency within the cells, impairing their ability to multiply and transform, thus acting as a potent barrier against tumor formation. This work reveals that some aspects of cellular aging can be protective, suggesting that therapies aimed at rejuvenating aged stem cells must be carefully considered in the context of cancer risk.

### Genomic integrity and DNA damage response in aging

While the epigenome may be reversible, maintaining genomic integrity remains essential in the aging process. Jan Hoeijmakers (Erasmus Medical Center, Rotterdam, Netherlands) presented data showing that accumulating DNA damage causes a genome-wide “transcription stress,” where RNA polymerases stall on damaged DNA [5]. As DNA damage is random, this disproportionately silences long genes [6], a phenomenon that can explain up to 50% of the transcriptomic changes in aging organs, occurs in species ranging from worms to humans [7] and positions the unexpectedly long IGF-1 gene as a master DNA damage sensor.

Vanessa Sinatti (Aptah Biosciences, San Carlos, CA, USA) presented a platform targeting the U1 snRNP complex, a complex essential for safeguarding the transcriptional integrity of long genes found in the brain and retina. This complex becomes dysfunctional and aggregates in the cytoplasm in Alzheimer’s disease (AD), and potentially during aging, often appearing even before tau pathology [8]. Aptah Bio’s “RNA WiCo” technology acts as a scaffold to restore U1 function. In AD patient neurons, their compound restored electrical activity, and in aging mice it reduced amyloid-beta and phospho-tau. Sinatti also showed that the compound had superior anti-tumor efficacy over the standard of care in glioblastoma xenograft models.

Complementing this, Björn Schumacher (University of Cologne, Cologne, Germany) emphasized the genome’s unique, irreplaceable role in aging [9]. His lab developed a precise transcriptomic aging clock in C. elegans capable of predicting a young worm’s lifespan [10]. Single-cell analyses revealed that neuron types within the same animal age at different rates, with environmentally exposed neurons aging fastest. These conserved neuronal aging signatures enabled an *in silico* screen of FDA-approved drugs, identifying novel neuroprotective candidates [11].

What dictates the rate of this deterioration? Zane Koch (UC San Diego, La Jolla, CA, USA) discussed a potential master regulator: the DREAM complex, a transcriptional repressor of DNA repair pathways [12]. Across 93 species, from the short-lived to the long-lived, he and his colleagues found a significant negative correlation: species with lower DREAM activity, like humans, live longer [13]. Complementing these findings, Anna Murray (University of Exeter, Exeter, UK), showed that the rate of reproductive aging is also substantially influenced by DNA repair. Her analysis of over 200,000 women found that nearly two-thirds of the 290 genetic signals linked to the timing of menopause are in DNA damage response genes [14]. This work supports the view that ovarian aging is primarily driven by declining genomic maintenance.

Further challenging old dogmas, Ben Shenhar (Weizmann Institute of Science, Rehovot, Israel) argued that the heritability of human lifespan has been greatly underestimated. By correcting for extrinsic mortality (like accidents or infections) from historical data, his group suggests that the heritability of our intrinsic lifespan is around 50%, placing it on par with other major human traits [15]. A key source of this heritable, age-related damage may lie in the “dark genome,” as proposed by Andrei Seluanov (University of Rochester, Rochester, NY, USA). He demonstrated that the age-related activation of transposable elements triggers chronic inflammation via the cGAS-STING pathway [16]. Mice engineered to repress these transposable elements were leaner, less inflamed, and dramatically less frail in old age, prompting Seluanov to propose that existing HIV drugs that inhibit reverse transcriptase could be repurposed to achieve a similar anti-aging effect in humans [17].

## Inflammaging and metabolic dysfunction

Chronic, low-grade inflammation, or “inflammaging”, was consistently identified as a key driver of aging. Miriam Merad (Precision Immunology Institute, New York, NY, USA) argued that macrophages are central mediators [18], showing that with age, protective tissue-resident macrophages are replaced by damaging, short-lived ones. Her lab found that administering spermidine, hypothesized to induce autophagy and limit monocyte-derived macrophage infiltration, restores the protective macrophage pool in aged mice, improving their immune responses. At the protein level, Andrea Heinz (University of Copenhagen, Copenhagen, Denmark) described elastin as a “non-renewable resource” synthesized only in early life. Its lifelong degradation not only causes loss of tissue function but releases inflammatory fragments called “elastokines,” creating a self-amplifying loop of damage [19, 20].

### Neuroinflammation and brain resilience

Neuroinflammation creates localized pockets of pathology. Using high-resolution biology, Marissa Schafer (Mayo Clinic, Rochester, MN, USA) used spatial molecular imaging to map the aging brain. Her team discovered that specific clusters of senescent, “disease-associated” microglia accumulate in the white matter of aged female mice, forming a self-reinforcing network of neuroinflammation with other senescent-like cells [21]. This aligns with growing evidence that senescent cells are far more heterogeneous than previously recognized, encompassing both pathological forms, such as the chronic, slowly accumulating senescence associated with aging tissues, and physiological forms serving beneficial roles. Tomaz Rozmaric (Ludwig Boltzmann Institute for Traumatology, Vienna, Austria) illustrated this distinction, showing that skin injury triggers a rapid senescence response within just 15 minutes, driven by translation of pre-existing p21 mRNA [22]. Rather than contradicting the slow accumulation seen in aging, this finding suggests that the kinetics of senescence are context- and cell type-specific, with acute, wound-induced senescence being an essential component of normal tissue repair.

Andy P. Tsai (Stanford University, Stanford, CA, USA) explored the unique resilience of the cerebellum, a brain region that shows early signs of molecular aging but is largely spared from Alzheimer’s pathology. He found that cerebellar microglia adopt a more neuroprotective state with age compared to those in the hippocampus. Spatial transcriptomics revealed a novel “neuronal-associated microglia” state, which appears when microglia are in direct contact with granule cells, suggesting this interaction shapes their protective function [23].

Lida Katsimpardi (INSERM; University of Montpellier, Montpellier, France) provided compelling evidence for the rejuvenating power of systemic factors on the brain, showing that young blood exposure can rejuvenate aged mice by increasing neural stem cells, remodeling vasculature, and improving cognitive function [24]. She identified the circulating factor GDF11 as a key player that improves memory when administered by stimulating neuronal activity, autophagy, and reducing hippocampal senescence [25]. Her team is also investigating how conditions like depression accelerate brain aging, noting that senolytics may reverse these effects [26]. They are additionally using organ-on-a-chip models to examine how the blood–brain barrier ages.

### Metabolic collapse and failure of quality control

In the brain, neuroinflammation manifests as metabolic collapse. Nobel laureate Morten Meldal (University of Copenhagen, Copenhagen, Denmark) presented a chemist’s approach to AD, designing “beta-bodies” to physically cap growing amyloid-beta (Aβ) fibrils. His lab also found that once Aβ peptides aggregate, they can catalyze their own further processing, turning the aggregated state itself into a toxic driver [27]. In the context of Parkinson’s disease, Michela Deleidi (Institute Imagine, Paris, France) used advanced human midbrain organoids to show that alpha-synuclein pathology causes a specific energy collapse in the most vulnerable neurons by disrupting mitochondrial NAD+ synthesis [28]. Riekelt Houtkooper (University of Amsterdam, Amsterdam, The Netherlands) provided a more nuanced view of this process, showing that while NAD+ levels drop significantly in the skeletal muscle of older adults [29], they remain stable in whole blood, questioning the use of blood NAD+ as a reliable biomarker, and calling for more clinical studies to strengthen the evidence for a role of NAD+ in human aging [30].

Ana Maria Cuervo (Albert Einstein College of Medicine, New York, NY, USA) detailed her work on chaperone-mediated autophagy (CMA), a selective lysosomal protein degradation pathway that declines with age. Using a novel fluorescent reporter mouse, her lab mapped CMA activity system-wide and showed overall CMA decline with age more pronounced in males [31]. They also found profound sex differences in what proteins are degraded by CMA in neurons [32]. Her team also developed a “CMA score” for humans, finding that it is low in patients with AD. Her lab has also developed small-molecule CMA activators [33] that successfully clear tau pathology and improve cognition in animal models of dementia [32].

Evandro Fang (University of Oslo, Oslo, Norway) argued that a decline in mitophagy, the recycling of damaged mitochondria, is a key driver of AD, leading to the buildup of A-beta and tau [34]. His lab found that the autophagy protein ULK1 is progressively reduced in AD patients. He demonstrated that NAD+ precursors and Urolithin A can restore mitophagy and improve memory in AD animal models, partly by reactivating autophagy genes via an NAD+-Sirt1-REST pathway. This work is now moving into clinical trials, including a Phase 2 trial for Urolithin A in AD patients [35].

This metabolic vulnerability is exacerbated by a mechanism highlighted by Luigi Ferrucci (National Institute on Aging, Baltimore, MD, USA): a systemic state of chronic cellular hypoxia driven by vascular and mitochondrial decline. He demonstrated that age-related vascular decline starves tissues of oxygen, accelerating epigenetic aging in mice, one such example of how the failure of one system can drive aging across the entire body [36]. Finally, Anne Brunet (Stanford University, Stanford, CA, USA) showcased her lab’s use of the turquoise killifish and longitudinal multi-omics to move beyond static snapshots. Her work maps the dynamic, interwoven trajectories of an individual’s lifespan, asking a critical new question: can we identify the tipping points where systemic decline becomes irreversible? [37].

### Can we predict the future? The quest for high-resolution clocks and biomarkers

To intervene in aging, we must first learn to measure it with precision. The conference showcased an explosion of new tools moving beyond chronological age to quantify biological age with unprecedented resolution, asking not just “how old are you?” but “how fast are you aging,” and “in which tissues?”.

A major leap forward is the development of organ-specific clocks. Tony Wyss-Coray (Stanford University, Stanford, CA, USA) demonstrated that by using plasma proteomics, his team can calculate an “age gap” for individual organs. These gaps are highly predictive: an accelerated “brain age” forecasts AD risk 15 years later, while a signature of synaptic proteins in the blood outperforms traditional biomarkers for predicting cognitive decline [38]. Similarly, Vadim Gladyshev (Harvard Medical School, Boston, MA, USA) developed proteomic clocks that predict organ-specific diseases and mortality, and reported the discovery that menopause triggers an abrupt, multi-organ acceleration of biological age [39]. Diving deeper into metabolism, Peter Mullen (University of Southern California, Los Angeles, CA, USA) profiled 12 different organs in mice to reveal that each tissue has a unique metabolic aging trajectory, allowing for the creation of clocks that identify key metabolic drivers of age in specific locations [40].

Epigenetic clocks are becoming more sophisticated. Steve Horvath (Altos Labs, San Diego, CA, USA) highlighted a new algorithm that can computationally dissect bulk tissue data to estimate the epigenetic age of specific cell types. When applied to the brains of patients with AD, it revealed that the age acceleration signal was coming specifically from neurons, a finding previously obscured in bulk tissue analyses [41]. In a novel visual approach, Alexey Terskikh (University of Western Australia, Perth, WA, Australia) introduced a method to capture the “epigenetic face” of a single cell [42]. Instead of sequencing, his lab uses 3D imaging of histone modifications, creating a visual biomarker that can track aging trajectories in immune cells as well as predict hearing loss by mapping an epigenetic gradient in the cochlea. Adding another layer of resolution, Christoph Kuppe (RWTH Aachen University, Aachen, Germany) used spatial multi-omics to map the aging human heart, identifying a previously unknown population of polyploid cardiomyocytes that appears in diseased hearts and drives harmful tissue remodeling [43].

### Translating clocks into actionable clinical biomarkers

Translating these clocks to the clinic is the next frontier. Ryan Smith (TruDiagnostic, Lexington, KY, USA) presented landmark data from human trials showing that statins can slow the DunedinPACE clock, while GLP-1 agonists like semaglutide can significantly reverse GrimAge in HIV patients [2, 44]. However, he also issued a critical warning of a “reproducibility crisis” due to technical inconsistencies, introducing a new unified microarray to standardize the field. Looking beyond the lab, researchers are developing more accessible biomarkers. Morten Scheibye-Knudsen (University of Copenhagen, Copenhagen, Denmark) demonstrated that computer vision is a powerful tool to measure effects of aging, for instance histological images can predict future cancer risk [45] and a simple facial photograph is a more powerful predictor of mortality upon hospital admission than any standard clinical test. Meanwhile, Jing-Dong Jackie Han’s lab (Peking University, Beijing, China) is using AI to develop novel clocks, including a single-cell morphological clock called “Morpho-Age,” which they used to screen thousands of chemicals for rejuvenation potential. Alexander Tyshkovskiy (Harvard Medical School, Boston, MA, USA) presented a suite of universal, interpretable transcriptomic aging clocks trained across multiple mammalian species [46]. His novel “mortality clocks” successfully track both aging and the effects of lifespan-modulating interventions, even at the single-cell level.

The quest for reliable biomarkers of aging is rapidly advancing, moving from broad systemic measures to more specific and actionable diagnostics. Nathan LeBrasseur (Mayo Clinic, Rochester, MN, USA) presented his work on developing a panel of circulating biomarkers of cellular senescence [47]. This panel, composed of proteins secreted by senescent cells, has proven to be a powerful predictor of a wide range of age-related health outcomes, including mobility disability, various chronic diseases, and mortality in older adults. His research has shown that these biomarkers are responsive to interventions like caloric restriction and exercise [48] and can help identify individuals who are most likely to benefit from senolytic therapies. LeBrasseur’s team is now leveraging advanced proteomics to discover new, organ-specific biomarkers of senescence, with the goal of creating more tailored diagnostics to assess the efficacy of interventions for specific conditions like idiopathic pulmonary fibrosis or heart failure.

Brian K. Kennedy (National University of Singapore, Singapore, Singapore) discussed the development and application of novel aging clocks. He presented data from a large community-based study that used a DNA methylation clock to assess the effects of various supplements on biological age. His findings suggest that certain interventions, such as time-released alpha-ketoglutarate, may have a significant impact on this biomarker [49]. Kennedy, in collaboration with Jan Gruber and Fong Sheng (National University of Singapore, Singapore, Singapore), also introduced a clinical chemistry-based clock that predicts mortality and functional decline, offering a more actionable tool for clinicians. This clock can identify the specific physiological systems that are driving an individual’s accelerated aging, allowing for more personalized and targeted interventions. He emphasized that improving near-term performance is a practical way to engage people in longevity interventions, since feeling better quickly can motivate sustained lifestyle changes that ultimately support long-term healthspan.

Fabrizio d'Adda di Fagagna (AIRC Institute of Molecular Oncology, Milan, Italy) demonstrated the therapeutic potential of targeting the DNA damage response at telomeres with antisense oligonucleotide designed to inhibit this pathway to reduce senescence, improve hematopoietic stem cell function, and fitness in various mouse models, including geriatric mice [50]. In addition, he offered a thought-provoking perspective on the relationship between different aging clocks. He presented data showing that while telomere shortening in mice leads to severe age-related pathologies, it may not impact some widely used DNA methylation-based aging clocks. This suggests that multiple, parallel aging processes may occur in the body without always intersecting, and that a multi-faceted approach targeting different aspects of aging may be necessary for comprehensive rejuvenation.

### The cutting edge of skin diagnostics

On the cutting edge of skin aging diagnostics, Anastasia Georgievskaya (Haut.AI, Tallinn, Estonia) showcased the power of generative AI in creating photographic biomarkers of skin aging [51]. Her company has developed a suite of AI-powered tools that can analyze skin images to quantify a wide range of features, from wrinkles and pigmentation to redness and texture [52]. These tools can then be used to generate hyper-realistic simulations of how an individual’s skin will age under different conditions, such as with or without sun protection, or with changes in lifestyle. This technology is not only valuable for personalizing skincare recommendations but also for creating synthetic data to train more accurate and inclusive diagnostic models. By providing a visual representation of the aging process, Georgievskaya believes that AI can be a tool for educating consumers and motivating them to adopt healthier habits.

Cristiana Banila (Mitra Bio, London, UK) presented a non-invasive approach to tracking skin health through epigenetic biomarkers. Her company has developed a tape-stripping method to collect skin samples from the epidermis, from which they can extract high-quality DNA for methylation analysis [53]. Using these samples, they have built the largest known database of skin epigenetic data and have developed skin-specific aging clocks. These clocks can be used to assess the efficacy of various skin interventions, from topical treatments to laser therapies. Furthermore, Banila’s team is developing epigenetic biomarkers to predict development of specific skin phenotypes, such as wrinkles and hydration, which could provide more clinically relevant endpoints for dermatology trials.

Elias Bou Samra (L’Oréal Research and Innovation, Aulnay-sous-Bois, France) discussed the role of microRNAs as key epigenetic regulators in skin longevity. This research, conducted by Claire Marionnet’s team, identified specific microRNAs that are involved in skin regeneration and differentiation [54], as well as those that are dysregulated in response to sun exposure [55]. This research opens up new avenues for the development of targeted cosmetic and therapeutic interventions that can modulate microRNA expression to promote skin health and combat the signs of aging.

## From discovery to design: The next generation of longevity interventions

With a deeper understanding of mechanisms and more precise biomarkers, the field is moving from repurposing old drugs to de novo design of novel therapeutics.

### Targeting cellular senescence and inflammation

A key evolution is happening in the world of senolytics. Rather than simply killing senescent cells, researchers are developing more nuanced “senomorphics” that modulate their harmful secretions. Marco Demaria (European Institute for the Biology of Ageing, Groningen, The Netherlands) discovered that detrimental senescent cells rely on the purinergic receptor P2X1 to amplify their own inflammatory signaling [56]. An inhibitor of P2X1 acted as a potent senomorphic, quieting the inflammatory senescence-associated secretory phenotype (SASP) and improving healthspan in multiple animal models without killing senescent cells.

Further, Kotb Abdelmohsen (National Institute on Aging, Baltimore, MD, USA) in collaboration with Yuta Lee (Accelerated Biosciences, Philadelphia, PA, USA), presented findings on the use of secretomes from human trophoblast stem cells (hTSCs) as a potent senomorphic therapy [57]. These embryonic stem cells, ethically sourced from pre-placental tissue of ectopic pregnancies, release a cocktail of factors that can systemically suppress SASP. In preclinical studies, the administration of hTSC-derived secretome or isolated extracellular vesicles to senescent cells reduced the expression of key SASP factors like IL-6 and IL-8, and in aged mice, it lowered circulating levels of pro-aging factors such as GDF-15 and CXCL1. This work suggests that the hTSC secretome may work by restoring DNA repair mechanisms in senescent cells.

Laura Niedernhofer (University of Minnesota, Minneapolis, MN, USA) detailed a high-throughput screening platform to identify novel senolytics. Her team has uncovered several new classes of compounds with senolytic activity, including a novel lipid, epigenetic regulators like LSD1 and BET inhibitors, and a drug targeting mitochondrial function [58]. These discoveries have shown promise in preclinical models, reducing senescence markers, improving the epigenetic landscape, and enhancing physiological function in aged animals. This work underscores the interconnectedness of the hallmarks of aging, as targeting pathways like mitochondrial dysfunction or epigenetic alterations can effectively reduce the burden of senescent cells.

In a similar vein, In Hwa Jang (University of Minnesota, Minneapolis, MN, USA) presented her work on GDF3, a cytokine that promotes inflammation in the adipose tissue of aged mice. Her research revealed that GDF3, primarily secreted by inflammatory adipose tissue macrophages, acts through the SMAD2/3 signaling pathway to alter chromatin accessibility, pushing macrophages towards a more inflammatory state. Notably, genetic deletion or pharmaceutical inhibition of the GDF3-SMAD2/3 axis protected aged mice from endotoxemia-induced inflammation and mortality, a benefit not seen in young mice [59]. This age-specific effect highlights a promising new target for mitigating chronic inflammation in older individuals. From a different angle, Christiane Volbracht (H. Lundbeck A/S, Valby, Denmark) presented a focused knockdown screen to find new targets for tauopathies, identifying the deubiquitinating enzymes ubiquitin-specific proteases USP7 and USP10 as key regulators whose inhibition promotes the clearance of toxic tau aggregates [60].

### Generative AI in drug discovery and protein engineering

The power of generative AI is rapidly accelerating drug discovery. In a significant achievement, Fedor Galkin (Insilico Medicine, Abu Dhabi, United Arab Emirates) showcased how their AI platform identified a novel target [61] for Idiopathic Pulmonary Fibrosis (IPF), designed a drug for it [62, 63], and advanced it through a successful Phase 2 trial [64]. The drug, rentosertib, improved lung function in patients and Insilico Medicine is currently inspecting its geroprotective properties with proteomic aging clocks. Based on preliminary results, rentosertib’s trial provides a strong proof-of-concept for developing therapies that treat a specific disease while targeting fundamental aging processes. In an even more futuristic approach, Joe Betts-LaCroix (Retro Biosciences, Redwood City, CA, USA) detailed a collaboration with OpenAI, in which a generative AI model designed entirely new versions of the Yamanaka reprogramming factors. Notably, some of these AI-generated proteins significantly outperformed the originals in reprogramming efficiency, indicating a new application for AI as a creative partner in protein engineering.

### Novel chemistries and advanced cellular therapeutics

Xiaodong Liu (Westlake University, Hangzhou, China) addressed a persistent challenge in cellular therapy: iPSCs (induced pluripotent stem cells) frequently retain residual epigenetic memory from their somatic cell of origin, compromising differentiation fidelity, functional maturity, and therapeutic consistency. His team developed a “Transient Naive Treatment” (TNT) strategy, demonstrating that a brief, defined 5-day exposure to naïve pluripotency conditions is sufficient to erase this residual epigenetic signature [65]. This TNT method produces iPSCs that are epigenetically and functionally superior, more equivalent to embryonic stem cells. The lab is now advancing this technology toward the clinic with an autologous iPSC-derived neuron therapy for Parkinson’s disease, which has completed pre-clinical safety and efficacy studies.

Restoring youthful metabolism remains a prime target. Eric Verdin (Buck Institute, Novato, CA, USA) argued against simple NAD+ supplementation, proposing instead to “plug the leak” of NAD+ consumption driven by the enzyme CD38. His lab identified the choroid plexus as a critical hotspot for age-related CD38 expression, linking it to blood-brain barrier dysfunction and neuroinflammation. To counter this, Verdin presented NTX748, a novel and highly potent CD38 inhibitor that restored NAD+ levels, enhanced neuronal plasticity (LTP), and reversed cognitive deficits in aged mice, with Phase 1 clinical trials planned for the near future [66].

Complementing this, Javier María Peralta Ramos (Weizmann Institute of Science, Rehovot, Israel) identified CD38 as an immunometabolic checkpoint on dysfunctional T-cells in individuals with familial AD that, when targeted in an AD mouse model, restored metabolic fitness, improved cognitive performance and reduced neuroinflammation [67]. The focus on female reproductive aging as a key intervention point was highlighted by Yousin Suh (Columbia University, New York, NY, USA), who is leading the VIBRANT clinical trial to test whether the mTOR inhibitor rapamycin can extend reproductive lifespan in women [68]. Broadening the intervention landscape, Edward Rudnic (Maxwell Biosciences, Austin, TX, USA) introduced CLAROMER^®^, synthetic molecules that mimic innate immune peptides to eliminate multi-drug-resistant infections without harming the gut microbiome, which he termed an “anti-aging bioreactor.”

### Are we more than our genes? The dialogue between lifestyle and our internal clocks

The systemic benefits of physical activity were a major focus. Bente Klarlund Pedersen (University of Copenhagen, Copenhagen, Denmark), a pioneer in the field, described how contracting muscle acts as an endocrine organ, secreting hundreds of “myokines” that directly counteract the process of inflammaging [69]. Conversely, her colleague Charlotte Suetta (University of Copenhagen, Copenhagen, Denmark), whose talk “Lying Still, Falling Fast” detailed how physical inactivity, such as during hospitalization, can trigger a rapid acceleration of muscle loss and aging [70]. Lykke Sylow (University of Copenhagen, Copenhagen, Denmark) highlighted the emerging concern that popular GLP-1-based weight-loss drugs can cause significant muscle loss, underscoring the need for interventions like exercise to preserve muscle and metabolic health [71].

Diet is also a powerful lever in aging. The work of Dudley Lamming (University of Wisconsin-Madison, Madison, WI, USA) challenged the “calorie is a calorie” paradigm, showing that restricting a single amino acid, valine, conferred many of the same healthspan benefits as caloric restriction in mice of both sexes, and extended male but not female lifespan [72]. In large human cohorts, Marta Guasch-Ferré (University of Copenhagen, Copenhagen, Denmark) found that adherence to healthy, plant-based dietary patterns was associated with up to an 86% greater chance of healthy aging, while consumption of ultra-processed foods was linked to significantly lower odds [73].

### New models and computational approaches

The development of new tools and models accelerates progress in aging research, enabling researchers to move beyond traditional methods to embrace biological complexity. Steven Austad (University of Alabama, Birmingham, AL, USA) advocated for using diverse animal models, including long-lived species and companion animals, over homogenous, short-lived lab mice to improve preclinical translation [74]. Emma Teeling (University College Dublin, Dublin, Ireland) shared her research on the longest-lived mammals for their size: bats. Her team found that long-lived bats defy the typical hallmarks of aging: their telomeres don’t shorten, they upregulate DNA repair with age, and they actively dampen inflammation [75]. Dario Valenzano (Leibniz Institute on Aging, Jena, Germany) uses the short-lived turquoise killifish to model brain aging. His work revealed that intracellular Amyloid beta (Aβ) drives cognitive decline in these fish, and knocking out the precursor protein rescued deficits, suggesting a target for preserving cognitive healthspan [76]. Adding another perspective, Kazuto Kawamura (Max Planck Institute for Biology of Ageing, Cologne, Germany) showed that in C. elegans, the biological age increase induced by starvation is dramatically reversed upon refeeding, a process regulated by the linker histone H1-0 [77]. This suggests that age is more fluid than previously thought.

Todd White (Thalion Initiative, Boston, MA, USA) argued that low preclinical drug success rates (1.2%) stem from insufficient fundamental biological understanding. He described Thalion as an entity funding large-scale projects to generate foundational knowledge. White noted that poor-quality, siloed data limits AI utility. To address this, one of Thalion’s projects, the “Biobank of Mammalian Species,” a $100 million effort, samples 200 mammalian species to provide deep multi-omics open-access data.

Technology for testing interventions is also evolving. Martin Borch Jensen (Gordian Biotechnology, San Francisco, CA, USA) showcased a platform that bypasses artificial lab models entirely by screening hundreds of gene therapies at once, directly *in vivo* in aged animals with naturally occurring diseases, such as osteoarthritic horses, and a developed disease-modifying osteoarthritis therapy [78]. This approach bridges the gap between discovery and real-world complexity. Rafael de Cabo (National Institute on Aging, Baltimore, MD, USA) presented the Study of Longitudinal Aging in Mice, a large-scale project designed to systematically test interventions in genetically diverse mouse populations to build a predictive framework for translation [79].

Computationally, the field is moving from data analysis to causal reasoning. Kennedy Schaal (Rejuve Bio, Orange, CA, USA) introduced a neuro-symbolic AI that reasons over a “knowledge metagraph” to generate testable, evidence-based hypotheses about aging. Going a step further, Michael Antonov (Deep Origin, Palo Alto, CA, USA) outlined a vision for building an integrated “virtual cell” that combines AI with physics-based simulations to create more robust models for drug discovery.

Maxim Kholin (Gero, Singapore) presented Peter Fedichev's gerophysics theory, which defines aging as resilience loss [80, 81], suggesting that interventions should halt the rate of loss rather than reverse damage to yield the greatest gains in lifespan extension. Paul Kohlhaas (Molecule AG, Basel, Switzerland) discussed Decentralized Science, proposing web3 technologies to create open markets for scientific intellectual property.

### From the science of longevity to the medicine of tomorrow

A consensus emerged on strategies to bring anti-aging drugs to market. Sergey Jakimov (Longevity Vision Fund, London, UK) urged researchers to build companies around specific diseases with clear regulatory paths. This disease-first strategy was perfectly exemplified by Insilico Medicine’s drug for IPF [64]. As Alex Zhavoronkov (Insilico Medicine, Cambridge, MA, USA) and Fedor Galkin explained, by achieving a clear clinical endpoint in a devastating disease while also demonstrating a reversal of aging biomarkers, they have created a new template for the field.

Jeroen Aerssens (Rejuvenate Biomed, Diepenbeek, Belgium) provided an update on RJx-01 [82], a combination therapy for sarcopenia that not only improved muscle strength in a Phase 1b trial but also reversed molecular signatures of aging in biopsies, with a Phase 2 trial now underway. Furthering the therapeutic pipeline, Mourad Topors (Repair Biotechnologies, Syracuse, NY, USA) presented a novel strategy for atherosclerosis that targets the buildup of free cholesterol within the liver, a “cholesterol curse”, which hinders the body’s ability to regress atherosclerotic plaque [83] while Rusty Montgomery (BioAge Labs, Richmond, CA, USA) showcased the clinical development of novel NLRP3 inflammasome inhibitors to directly combat inflammaging [84].

Lotte Bjerre Knudsen (Novo Nordisk, Måløv, Denmark) provided a compelling perspective on how a single therapeutic innovation can transform an entire field. She described the journey of GLP-1 receptor agonists, originally developed for diabetes and obesity, but now also recognized for their profound cardiovascular, liver and renal protective effects, as a clear example of how disease-targeted drugs can evolve into systemic, healthspan-promoting therapies [85].

Nobel laureate Michael Levitt (Stanford University, Stanford, CA, USA) analyzed the COVID-19 pandemic as a stress test exposing societal vulnerabilities [86]. Addressing institutional bottlenecks, Niklas Anzinger (Infinita City, Prospera, Roatán, Honduras) cited Eroom’s Law and the regulatory path itself holds back research and clinical development. Niklas’ organization is working directly on alternative regulatory pathways that allow more real-world data collection like the Prospera jurisdiction in Honduras, and Montana’s “Right to Try” bill SB 535 that allows the general population to access Phase 1-cleared therapies.

#### Bridging preclinical findings to human application

Translating basic aging research into clinical application remains a central challenge. John Newman (Buck Institute, Novato, CA, USA) addressed the challenge of translating basic aging research into clinical practice, describing his efforts to build a translational geroscience program bridging this gap. He highlighted the value of early proof-of-concept trials to de-risk interventions and validate preclinical mechanisms, using his research on ketone bodies as an example [87]. Collin Ewald (Novartis, Cambridge, MA, USA) outlined the company’s strategy to develop regenerative pharmacological interventions targeting diseases of aging mechanisms to restore cell and tissue function in patients, guided by large-scale human data. He highlighted focus areas including mitochondrial health, directing cell fate and cellular rebalancing, DNA repair, extracellular matrix homeostasis, and exercise biology, with translational applications in indications such as neurodegenerative, cardiovascular, metabolic, and immune-related diseases [88]. Andrew Adams (Eli Lilly, Indianapolis, IN, USA) proposed shifting from “sick care” to “health care” via preventative medicines. He cited statins and GLP-1 agonists as examples, suggesting GLP-1 agonists could function as longevity drugs due to their effects on age-related conditions and all-cause mortality. He also emphasized Lilly’s commitment to early intervention in AD.

Finally, Weilan Wang (National University of Singapore, Singapore, Singapore) presented unpublished real-world data on gerotherapeutic drugs in geriatric rehabilitation inpatients. Metformin and ACE inhibitors were associated with significantly lower one-year mortality. Machine learning models suggested these effects may be causal, and drug combinations showed greater benefit than single drugs.

Jamie Justice (XPRIZE, Los Angeles, CA, USA) detailed the $101 million Healthspan competition, a “moonshot” demanding not just the slowing of aging, but a 10-year functional restoration across muscle, cognition, and immunity within a single year. With the top 40 teams now advancing, the competition is driving a wave of multimodal strategies that combine lifestyle interventions with therapeutics, ranging from rapamycin to novel senolytics, to meet these rigorous new clinical endpoints [89].

#### Commercial, investment, and collaborative strategies

Industry leaders agree on a “disease-first” strategy. Companies must target recognized illnesses like IDF or AD. Repurposing cheap, off-patent drugs like metformin fails mathematically. Phase 3 clinical trials cost hundreds of millions of dollars. Companies cannot recover this money without a patent monopoly. Therefore, the industry tests new, patented drugs for specific diseases. During these trials, researchers simultaneously measure aging biomarkers like epigenetic clocks. This secondary strategy generates the hard numbers of regulators demand. The goal is to force regulators to classify aging as a reimbursable medical condition. This mirrors how objective data transformed obesity from a lifestyle choice into a treated disease.

This regulatory path dictates how investors allocate money. A panel of top investors (Murali Venkatesan, Tom Zuber, Kitsu Egerton, and Daisy Cai) outlined the funding mechanics. Growth-stage capital funds safe, patented platforms. Investors buy portfolios of multiple drugs to avoid the binary risk of a single clinical failure. At the seed stage, investors judge founders entirely on execution speed. The primary barrier to market is insurance reimbursement, not science. Furthermore, medical diagnostics take 10 to 20 years to clear regulators. To survive this delay, capital flows into consumer-facing models. These models bypass doctors and insurers entirely. Founders must build a profitable business today to fund the ultimate goal of systemic aging intervention.

These strict financial rules force biotech startups to partner with massive pharmaceutical companies. Industry leaders (Lisa Melton, Avi Spier, Heinrich Jasper, Kristen Fortney, Aisyah Sjöholm, and Lars Hartenstein) defined this exchange. Large pharma companies use aging biology to find novel targets for existing diseases. This strategy limits their risk and guarantees reproducible data. Startups secure these lucrative partnerships by offering exclusive, long-term human datasets. Large companies need this human data to translate lab results into approved cures. Biological validity is no longer the bottleneck. The actual barrier is a missing “transportable narrative.” Founders lack a simple, compelling story to extract money from investors and governments. Current funding remains disproportionately low relative to the societal burden of aging-related diseases.

## Concluding vision and future directions

The 12th ARDD meeting was not merely a showcase of answers; it also generated new, more specific questions. The presentations collectively outlined a future where medicine is proactive rather than reactive, focused on maintaining youthful function rather than managing decline.

The new frontiers are clear. Can we develop a unified theory that connects the deterioration of the genome, the drift of the epigenome, and the loss of systemic resilience? How do we translate the dynamic, multi-omic maps of aging into personalized, combinatorial interventions that can steer an individual’s aging trajectory? What are the ethical frameworks required for a world where we can not only predict disease decades in advance but actively intervene to prevent it?

The research shared in Copenhagen signals that we are moving from the era of understanding aging to the era of engineering longevity (Figure 2). The ultimate challenge is no longer just to add years to life, but to add life to years, and—in doing so—to reimagine the very nature of human potential. The field is now equipped with new models, computational tools, and a clearer understanding of aging’s molecular drivers, positioning it for significant translational progress in the coming decade.
